# EZH2 as a prognostic-related biomarker in lung adenocarcinoma correlating with cell cycle and immune infiltrates

**DOI:** 10.1186/s12859-023-05271-7

**Published:** 2023-04-17

**Authors:** Kui Fan, Bo-hui Zhang, Deng Han, Yun-chuan Sun

**Affiliations:** 1 Department of Radiation Oncology, Cangzhou Hospital of Integrated Traditional Chinese and Western Medicine-Hebei Province, No. 31, Huanghe West Road, Yunhe District, Cangzhou, 061000 Hebei China; 2 Department of Neurology, Cangzhou Hospital of Integrated Traditional Chinese and Western Medicine-Hebei Province, Cangzhou, 061000 Hebei China; 3 Division of Gastroenterology, Dongzhimen Hospital, Beijing University of Chinese Medicine, Beijing, 100105 China

**Keywords:** EZH2, LUAD, Prognosis, Tumor microenvironment

## Abstract

**Backgrounds:**

It has been observed that high levels of enhancer of zeste homolog 2 (EZH2) expression are associated with unsatisfactory prognoses and can be found in a wide range of malignancies. However, the effects of EZH2 on Lung Adenocarcinoma (LUAD) remain elusive. Through the integration of bioinformatic analyses, the present paper sought to ascertain the effects of EZH2 in LUAD.

**Methods:**

The TIMER and UALCAN databases were applied to analyze mRNA and protein expression data for EZH2 in LUAD. The result of immunohistochemistry was obtained from the HPA database, and the survival curve was drawn according to the library provided by the HPA database. The LinkedOmics database was utilized to investigate the co-expressed genes and signal transduction pathways with EZH2. Up- and down-regulated genes from The Linked Omics database were introduced to the CMap database to predict potential drug targets for LUAD using the CMap database. The association between EZH2 and cancer-infiltrating immunocytes was studied through TIMER and TISIDB. In addition, this paper explores the relationship between EZH2 mRNA expression and NSCLC OS using the Kaplan–Meier plotter database to further validate and complement the research. Furthermore, the correlation between EZH2 expression and EGFR genes, KRAS genes, BRAF genes, and smoking from the Cancer Genome Atlas (TCGA) database is analyzed.

**Results:**

In contrast to paracancer specimens, the mRNA and protein levels of EZH2 were higher in LUAD tissues. Significantly, high levels of EZH2 were associated with unsatisfactory prognoses in LUAD patients. Additionally, the coexpressed genes of EZH2 were predominantly associated with numerous cell growth-associated pathways, including the cell cycle, DNA replication, RNA transport, and the p53 signaling pathway, according to Gene Ontology and Kyoto Encyclopedia of Genes and Genomes pathways. The results of TCGA database revealed that the expression of EZH2 was lower in normal tissues than in lung cancer tissues (*p* < 0.05). Smoking was associated with elevated EZH2 expression (*p* < 0.001). EZH2 was highly expressed in lung cancers with positive KRAS expression, and the correlation was significant in lung adenocarcinoma (*r* = 0.3129, *p* < 0.001). CMap was applied to determine the top 15 positively correlated drugs/molecules and the top 15 negatively correlated drugs/molecules. MK-1775, MK-5108, fenbendazole, albendazole, BAY-K8644, evodiamine, purvalanol-a, mycophenolic-acid, PHA-793887, and cyclopamine are potential drugs for patients with lung adenocarcinoma and high EZH2 expression.

**Conclusions:**

Highly expressed EZH2 is a predictor of a suboptimal prognosis in LUAD and may serve as a prognostic marker and target gene for LUAD. The underlying cause may be associated with the synergistic effect of KRAS, immune cell infiltration, and metabolic processes.

## Introduction

Globally, pulmonary carcinoma exhibits the highest incidence and mortality rate [1, 2]. Non-small cell lung cancer (NSCLC) accounts for between 80 and 85% of all pulmonary carcinoma [1]. Recent advances in the diagnosis and treatment of NSCLC have been remarkable, however, the 5-year overall survival (OS) rate remains below 21% [2]. The subtypes of NSCLC include squamous cell carcinoma, adenocarcinoma, and large cell carcinoma [3]. Lung adenocarcinoma is the most prevalent type, accounting for approximately 40% of lung cancers [4]. Surgery with radiotherapy significantly improves the 5-year survival rate for patients with early-stage lung adenocarcinoma [5]. For patients with early-stage lung adenocarcinoma, surgery with radiotherapy significantly improves the 5-year survival rate (83%, 68%, 60%, and 53% for stage IA, IB, IIA, and IIB patients, respectively, based on clinical stage staging). Patients with advanced lung adenocarcinoma are, however, rarely treated using the same methods. Their 5-year survival rate is approximately 10%, therefore, immunotherapy and targeted therapy for lung cancer have become a hot topic of research in recent years [6, 7]. In the first-line metastatic setting for patients with metastatic LUAD, immunotherapy-based combinations are considered significant breakthroughs and demonstrate efficacy and OS benefits [8]. Even with its success, only a subset of patients exhibited responses, hence necessitating the development of prediction markers. Numerous studies further elucidate subsequent tumor progression and therapeutic response in relation to oncocyte and tumor microenvironment (TME) interactions [9].

There is a growing body of evidence suggesting that epigenetic variations facilitate cancer developmental processes and treatment reactions. Through DNA methylation and demethylation, histone modification, and chromatin remodeling, epigenetic modifications can modulate chromatin status and genetic expression [10–12] without affecting DNA sequences. Enhancer of zeste homolog 2 (EZH2) is a gene associated with conservative cellular bio functions (e.g., cellular cycle, cellular proliferative ability, and cellular differentiative activity). EZH2 is essential for the proliferation and metastasis of cancer [13–15]. In vitro and in vivo knockdown of CBX2 significantly inhibited the growth and metastasis of LUAD cells with high EZH2 expression. While the combination of high CBX2 and EZH2 expression was indicative of an unfavorable prognosis for LUAD [16]. Highly expressed EZH2 is indicative of unsatisfactory prognoses for NSCLC, which may be associated with cancer phases or carcinoma types. EZH2 may be an independent NSCLC prognostic index. The prognosis of NSCLC [12] is influenced by highly expressed EZH2 or its synergy with KRAS or BRAF variants. EZH2 is a promising biomarker candidate with excellent immunotherapy response potential. Highly expressed EZH2 was associated with an unsatisfactory response to anti-PD-1 therapy, an early relapse, and death. In addition, the abnormal expressing level of EZH2 correlates with the sensitivity of cisplatin-based therapy [17].

Using multiple network databases, this paper conducted exhaustive and systematic analyses of the EZH2 expression level in LUAD. The authors found EZH2 to be significantly overexpressed in LUAD. EZH2 coexpression genes are predominantly abundant during cell development, T cell stimulation, and acquired immune responses. Both EZH2 expressions were related to cancer-infiltration immunocytes and immune modulators in LUAD. This study reveals that EZH2 is an underlying prognostic and predictive marker for response to treatment with immune-checkpoint inhibitors in NSCLC patients.

## Materials and methods

### Tumor immune estimation resource (TIMER) analyses

The TIMER (https://cistrome.shinyapps.io/timer/) online server is a comprehensive network for studying the interactions of immune infiltrates with multiple tumor types [20]. The team analyzed the RNA sequences of various cancer types in TCGA with the TIMER database to determine the differential expression of EZH2 in tumor and paracancerous tissues. The TIMER algorithm estimated the immune cell abundance. Association modules were applied to determine the relationship between the RNA sequencing expression profile data of EZH2 in LUAD and immunocytes, such as B Cell, CD8+ T cell, CD4+ T cell, Macrophage, Neutrophil, and Dendritic Cell. Based on genetic modules, the genetic biomarkers of immunocytes were also related to the expression level of EZH2. These genetic biomarkers have been mentioned in previously published articles [18–20].

### UALCAN analyses

UALCAN (http://ualcan.path.uab.edu/) is a comprehensive, facilitative, interaction-based online resource for the analyses of cancer omics data and tumor clinic information from TCGA [21]. In the UALCAN database, this study obtained EZH2 mRNA and protein that were differentially expressed in LUAD and adjacent healthy tissue samples.

### Human protein atlas (HPA) database analysis

To map tissue samples, cells, and organs, the HPA (https://www.proteinatlas.org/) is based on proteomics, transcriptomes, and system biology information. It contains the protein expression of tumor tissue and healthy tissue, as well as the survival curve for patients with tumors. The HPA database was queried for immunohistochemical data regarding EZH2 expression in LUAD. In addition, the survival data was applied to draw the survival curve.

### Linked omics data base analyses

The Linked Omics database (http://www.linkedomics.org/login.php) is an online platform for analyzing multiple omics data sets from the TCGA [22]. Using the Link Finder module, the database was screened for LUAD for DEGs related to EZH2. The Pearson correlative coefficient was utilized to analyze the correlational outcomes, which were represented by volcanic plots and heat maps. The DEGs associated with EZH2 were annotated with GO analyses, KEGG analyses [23–25], and GSEA through the Link Interpreter module in order to acquire descriptive data.

### Connectivity map (CMap) analyses

CMap (https://clue.io/) [26–28] can be utilized to discover the mechanism of action of small molecules, functionally annotate genetic variants of disease genes, and inform clinical trials. The CMap database was updated with the top 50 EZH2-related up- and down-regulated genes from The Linked Omics database in order to predict potential drug targets for LUAD. And these potential therapeutic targets are ranked according to a point system.

### TISIDB data base analyses

The TISIDB database (http://cis.hku.hk/TISIDB) is an online platform for analyzing the interaction between cancer and the immune system, which facilitates the prediction of immune therapy reactions [29]. Through using TISIDB database, this study examined the relationship between the expression level of EZH2 and lymph cells, immune modulators, and chemotactic factors. At *p* < 0.05, a ‘*rho*’ value > 0.2 and <  − 0.2 was deemed to indicate a significant association at *p* < 0.05 [30].

### Bioinformatics analysis based on TCGA database

To further validate and supplement our research, the relationship between EZH2 mRNA expression and NSCLC OS is analyzed with the Kaplan–Meier plotter database (https://kmplot.com) [31–33]. Lung adenocarcinoma and lung squamous cell carcinoma TCGA data on EZH2 transcriptome expression were retrieved from the TCGA data portal. In addition, we evaluated the correlation between EZH2 expression and the EGFR gene, the KRAS gene, the BRAF gene, and smoking in lung cancer patients from the TCGA database. When the *p*-value was less than 0.05, the results were deemed statistically significant.

### Statistics

The statistical analysis was performed using GraphPad Prism 8.0 (America) and SPSS 17.0. (America). The measuring data are displayed as average ± SD. The expression levels of EZH2 mRNAs were compared between LUAD and neighboring healthy specimens from the TCGA database utilizing a t-test on independent samples. The TIMER database algorithm was implemented to estimate the immunocyte density. Utilizing the association module, the relationship between RNA-seq expression profile data and immune cells of EZH2 in LUAD was assessed. The Pearson correlation coefficient was applied to observe the EZH2 genes with differential expression in LUAD. Kaplan–Meier (K–M) curves were applied to conduct OS analyses. A two-tailed *p* = 0.05 was statistically significant.

## Results

### EZH2 is expressed in LUAD and is associated with prognosis

**Fig. 1:**
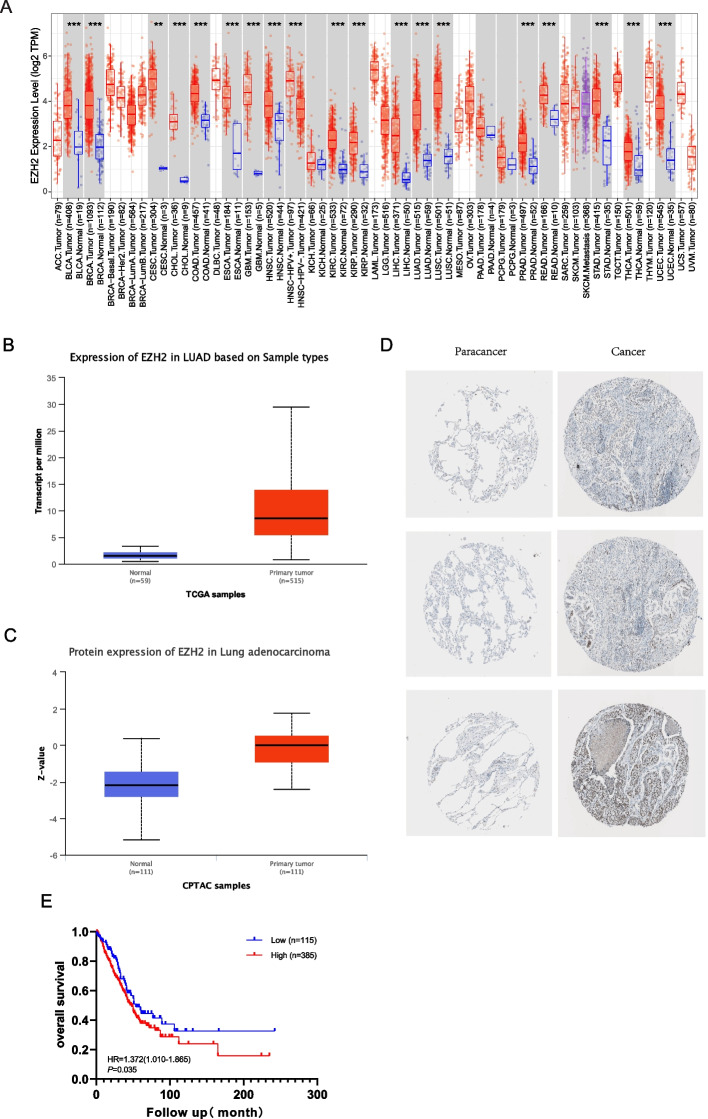
High expression of EZH2 in LUAD. **A** Human expression levels of EZH2 in various malignant tumor types from The Cancer Genome Atlas (TCGA) database were analyzed by the Tumor Immune Estimation Resource (TIMER). EZH2 was upregulated in Bladder Urothelial cancer (BLCA), mammary cancer (BRCA), Cervical squamous cell cancer and endocervical glandular cancer (CESC), biliary tract cancer (CHOL), Colonic glandular cancer (COAD), Esophagus cancer (ESCA), Glioblastoma multiforme (GBM), Head and Neck squamous cell cancer (HNSC), HNSC−HPV+, Kidney renal clear cell cancer (KIRC), Kidney renal papillary cell cancer (KIRP), Liver cell cancer (LIHC), Lung glandular cancer (LUAD), Lung squamous cell cancer (LUSC), Prostate glandular cancer (PRAD), Rectum glandular cancer (READ), Stomach glandular cancer (STAD), Thyroid cancer (THCA), Uterine Corpus Endometrial cancer (UCEC). *p < 0.05. **p < 0.01. ***p < 0.001. **B** The expressing levels of EZH2 mRNA in tumor specimens and healthy specimens were based on the UALCAN database. **C** The protein contents of EZH2 in paracancerous specimens and LUAD cancerous specimens were detected via CPTAC specimens in the UALCAN data base. **D** The representative EZH2 immunohistochemical images were found in LUAD cancer and corresponding normal tissues. **E** Kaplan–Meier survival analysis revealed that LUAD patients with high EZH2 expression exhibited a shorter overall survival than that in patients with low EZH2 expression

Compared with normal tissues, EZH2 was upregulated in Bladder Urothelial cancer (BLCA), mammary cancer (BRCA), Cervical squamous cell cancer and endocervical glandular cancer (CESC), biliary tract cancer (CHOL), Colonic glandular cancer (COAD), Esophagus cancer (ESCA), Glioblastoma multiforme (GBM), Head and Neck squamous cell cancer (HNSC), HNSC−HPV+, Kidney renal clear cell cancer (KIRC), Kidney renal papillary cell cancer (KIRP), Liver cell cancer (LIHC), Lung glandular cancer (LUAD), Lung squamous cell cancer (LUSC), Prostate glandular cancer (PRAD), Rectum glandular cancer (READ), Stomach glandular cancer (STAD), Thyroid cancer (THCA), and Uterine Corpus Endometrial cancer (UCEC) (Fig. 1A, B). Consistent with the data regarding the level of mRNA expression, our team discovered that the level of EZH2 protein expression was greater in LUAD specimens than in neighboring specimens (Fig. 1C). The positive dyeing of EZH2 was predominantly in the plasma and membranes (Fig. 1D). As illustrated by Fig. 1B, EZH2 was expressed more in cancer tissue than in paracancer tissue (Fig. 1B). K–M analyses revealed that high EZH2 expression was significantly associated with inferior OS [hazard ratio (HR) = 1.372, *p* = 0.035] in LUAD patients. The median value of EZH2 expressing level was 3.95. Patients with a higher expressing level had a 5-year OS of 38%, while those with a lower expressing level had a 5-year OS of 47% (Fig. 1E).

### EZH2 co-expression network in LUAD

**Fig. 2:**
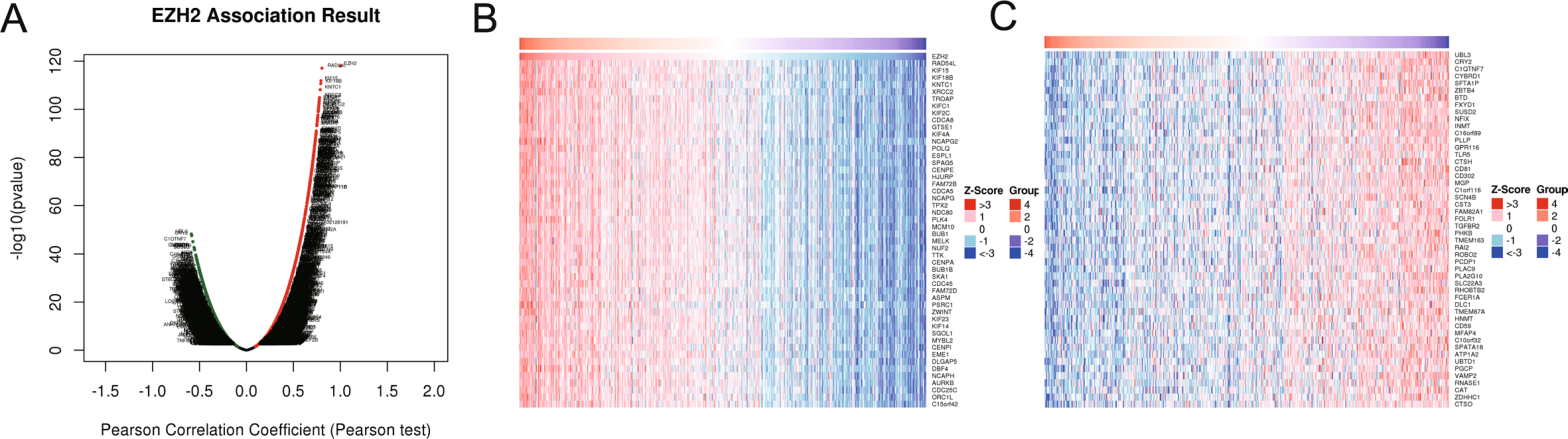
EZH2 co-expressed genes. **A** Volcanic plot of coexpressed profiling of EZH2 in LUAD via the Linked Omics data base. **B**, **C** Heatmaps of the 50 positively (**B**) and 50 negatively (**C**) related genes with EZH2 are presented

The findings of the coexpression feature of EZH2 revealed that 6559 genes were positively associated with EZH2, while 6209 genes were negatively associated (Fig. 2A). Positive and negative heatmaps displayed the top 50 genes related to EZH2 in a positive and negative manner, respectively (Fig. 2B, C).

GO analyses unveiled that coexpressed genes of EZH2 join predominantly in DNA replication, protein localization to the cell surface, adrenergic receptor signaling pathway, basal part of the cell, MHC protein complex, transcriptional factor (TF) activity, direct ligand modulated sequence-specific DNA binding, transmembrane receptor protein kinase activity, immunoglobulin binding, etc. (Tables 1, 2, 3).

**Table 1:** EZH2 co-expression genes were annotated by Biological Process (BP) analysis

Description	Size	Leading edge number	ES	NES	P value	FDR
DNA strand elongation	23	20	0.930	1.984	0	0
Interstrand cross-link repair	39	19	0.882	2.064	0	0
Chromosome segregation	262	99	0.874	2.525	0	0
DNA replication	233	101	0.853	2.461	0	0
Postreplication repair	46	16	0.849	2.062	0	0
Protein activation cascade	87	29	− 0.659	− 2.049	0	0
Protein localization to cell surface	56	26	− 0.698	− 2.033	0	0
Fluid transport	30	16	− 0.712	− 1.847	0	0
Response to fluid shear stress	33	14	− 0.716	− 1.881	0	0
Adrenergic receptor signaling pathway	28	13	− 0.739	− 1.903	0	0

*ES* enrichment score, *NES* Normalized Enrichment Score

**Table 2:** EZH2 co-expression genes were annotated by Cellular Component (CC) analysis

Description	Size	Leading edge number	ES	NES	P value	FDR
Condensed chromosome	192	82	0.881	2.538	0	0
Replication fork	62	36	0.877	2.178	0	0
Chromosomal region	288	117	0.859	2.544	0	0
Sex chromosome	29	9	0.848	1.860	0	0
Heterochromatin	72	26	0.808	2.085	0	0
Sarcolemma	130	51	− 0.608	− 2.050	0	0
Endosome lumen	35	6	− 0.617	− 1.684	0	0
Basal part of cell	49	19	− 0.691	− 1.966	0	0
Platelet dense granule	20	10	− 0.734	− 1.712	0	0
MHC protein complex	19	16	− 0.782	− 1.878	0	0

*ES* enrichment score, *NES* Normalized Enrichment Score

**Table 3:** EZH2 co-expression genes were annotated by Molecular Function (MF) analysis

Description	Size	Leading edge number	ES	NES	P value	FDR
Structural constituent of nuclear pore	22	11	0.819	1.765	0.000	0.002
Catalytic activity, acting on DNA	170	77	0.807	2.269	0.000	0.000
Single-stranded DNA binding	93	42	0.781	2.063	0.000	0.000
Helicase activity	142	53	0.779	2.168	0.000	0.000
Transcription factor activity, direct ligand regulated sequence-specific DNA binding	47	17	− 0.607	− 1.771	0.006	0.013
Oxidoreductase activity, acting on peroxide as acceptor	54	19	− 0.618	− 1.819	0.000	0.012
Transmembrane receptor protein kinase activity	80	30	− 0.631	− 1.972	0.000	0.000
Immunoglobulin binding	22	12	− 0.687	− 1.696	0.005	0.019
Catecholamine binding	19	8	− 0.745	− 1.770	0.009	0.012

*ES* enrichment score, *NES* Normalized Enrichment Score

KEGG analyses revealed the enriched pathways in the Cellular cycle, DNA replication, Homologous recombination, Fanconi anemia pathway, Spliceosome, Mismatch repair, RNA transportation, Nucleotide excision repair, p53 signaling pathway, Oocyte meiosis, Metabolism of xenobiotics by cytopigment P450, PPAR signal path, Cellular adhesive molecules (CAMs), Chemical tumorigenesis, Staphylococcus aureus infection, Salivary secretion, Retinol metabolic process, Lysosome, Complement and coagulation cascades, Drug metabolic process, etc. (Table 4). These findings indicate that the net of EZH2 expressing significantly affects the immune microenvironment in LUAD, and that the net of EZH2 expressing is essential for the onset and progression of cancers.

**Table 4:** EZH2 co-expression genes were annotated by Kyoto Encyclopedia of Genes and Genomes (KEGG) pathway analysis

Description	Size	Leading edge number	ES	NES	P value	FDR
Cell cycle	118	54	0.867	2.351	0	0
DNA replication	36	26	0.930	2.199	0	0
Homologous recombination	34	23	0.904	2.065	0	0
Fanconi anemia pathway	44	25	0.887	2.063	0	0
Spliceosome	115	71	0.740	2.009	0	0
Mismatch repair	23	14	0.922	1.977	0	0
RNA transport	158	64	0.689	1.933	0	0
Nucleotide excision repair	45	13	0.787	1.926	0	0
p53 signaling pathway	69	14	0.723	1.888	0	0
Oocyte meiosis	118	30	0.691	1.871	0	0
Metabolism of xenobiotics by cytochrome P450	70	23	− 0.638	− 1.942	0	0
PPAR signaling pathway	74	22	− 0.635	− 1.955	0	0
Cell adhesion molecules (CAMs)	137	56	− 0.595	− 1.990	0	0
Chemical carcinogenesis	75	21	− 0.667	− 2.009	0	0
Staphylococcus aureus infection	52	28	− 0.697	− 2.020	0	0
Salivary secretion	85	25	− 0.639	− 2.022	0	0
Retinol metabolism	63	22	− 0.669	− 2.025	0	0
Lysosome	121	52	− 0.620	− 2.039	0	0
Complement and coagulation cascades	78	25	− 0.656	− 2.049	0	0
Drug metabolism	66	23	− 0.713	− 2.160	0	0

*ES* enrichment score, *NES* Normalized Enrichment Score

### EZH2 Is related to immunocyte infiltration in LUAD

**Fig. 3:**
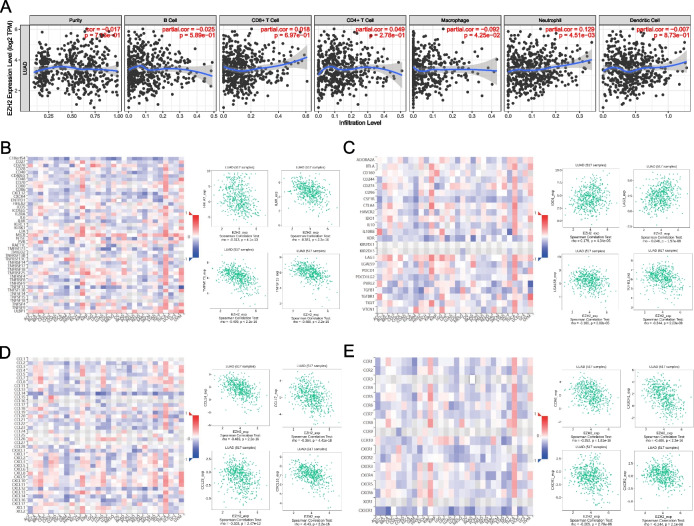
Association between EZH2 with immunocyte infiltration in LUAD. **A** Association between the expressing level of EZH2 and the richness of cancer-infiltrating immunocytes in LUAD based on the TIMER data base. **B**, **C** Based on the TISIDB database, the association between the expressing level of EZH2 in LUAD and immune stimulators (**B**) and immune inhibitors (**C**) was obtained. **D**, **E** Based on the TISIDB database, the association between the expressing level of EZH2 in LUAD and chemokines (**D**) and chemokine receptors (**E**) was obtained

EZH2 was positively correlated with neutrophil infiltration (*pr* = 0.129, *p* = 4.51e−03) and a negative correlation with macrophage (*pr* =  − 0.092, *p* = 4.25e−02) (Fig. 3A). EZH2 was remarkably related to immunostimulators, such as HHLA2 (*rho* =  − 0.313, *p* = 4.1e−13), IL6R (*rho* = −0.381, *p* < 2.2e−16), TMEM173 (*rho* = −0.499, *p* < 2.2e−16), and TNFSF13 (*rho* = -0.488, *p* < 2.2e−16) (Fig. 3B). The expressing level of EZH2 was associated with immune inhibitors such as IDO1(*rho* = 0.179, *p* = 4.34e−05), LAG3(*rho* = 0.246, *p* = 1.57e−08), LGALS9(*rho* = −0.186, *p* = 2.08e−05), and TGFB1(*rho* = −0.244, *p* = 2.23e−08) (Fig. 3C). The level of EZH2 expression was significantly correlated with CCL14 (*rho* = − 0.483, *p* = 2.2e−16), CCL17 (*rho* = − 0.364, *p* = 4.41e−18), CCL23 (*rho* = − 0.303, *p* = 2.47e−12), and CXCL16 (*rho* = − 0.43, *p* = 2.2e−16) (Fig. 3D). Moreover, the level of EZH2 expression was significantly correlated with chemotactic factor acceptors CCR6 (*rho* = − 0.352, *p* = 1.81e−16), CX3CR1 (*rho* = − 0.486, *p* < 2.2e−16), CXCR1 (*rho* = − 0.205, *p* = 2.78e−06), CXCR2 (*rho* = − 0.244, *p* = 2.1e−08) (Fig. 3E). These results validate the hypothesis that EZH2 is an immune regulatory factor in LUAD.

### EZH2 expression analysis through TCGA database

**Fig. 4:**
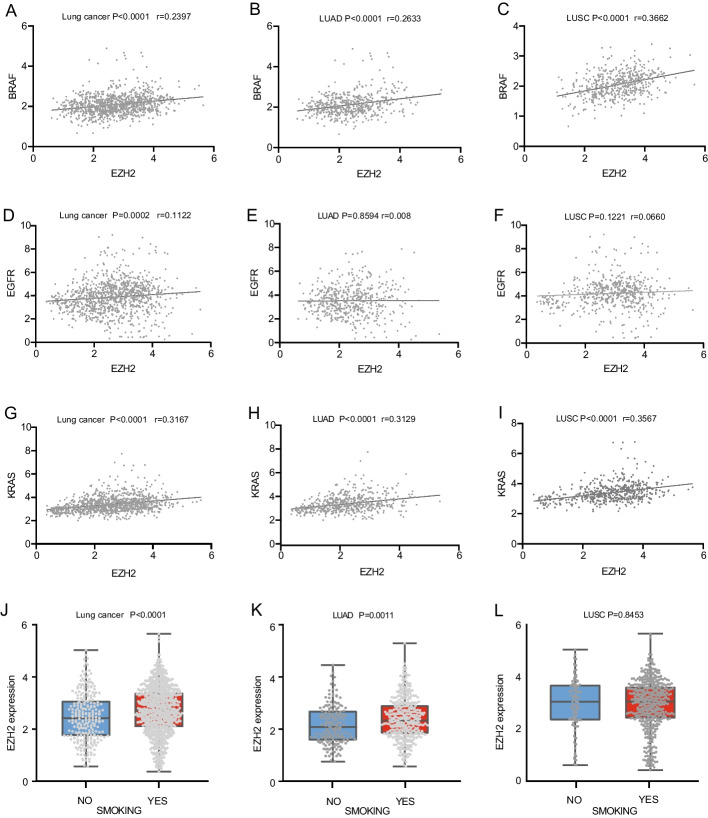
The TCGA data analysis. **A** Overall correlation between EZH2 and BRAF in adenocarcinoma and squamous cell carcinoma. **B** Correlation between EZH2 and BRAF in adenocarcinoma. **C** Correlation between EZH2 and BRAF in squamous cell carcinoma. **D** Overall correlation between EZH2 and EGFR in adenocarcinoma and squamous cell carcinoma. **E** Correlation between EZH2 and EGFR in adenocarcinoma. **F** Correlation between EZH2 and EGFR in squamous cell carcinoma. **G** Overall correlation between EZH2 and KRAS in adenocarcinoma and squamous cell carcinoma. **H** Correlation between EZH2 and KRAS in adenocarcinoma. **I** Correlation between EZH2 and KRAS in squamous cell carcinoma. **J** Overall correlation between EZH2 and Smoking in adenocarcinoma and squamous cell carcinoma. **K** Correlation between EZH2 and Smoking in adenocarcinoma. **L** Correlation between EZH2 and Smoking in squamous cell carcinoma. *Noted*: Lung Cancer: adenocarcinoma of lung and squamous cell lung carcinoma. *LUAD* adenocarcinoma of lung, *LUSC* squamous cell lung carcinoma, *r* coefficient of correlation

A total of 165 non-smokers and 361 smokers were extracted from the TCGA database of lung adenocarcinoma patients; 550 lung adenocarcinoma patients and 550 lung squamous carcinoma patients with EGFR expression; 526 lung adenocarcinoma patients and 550 lung squamous carcinoma patients with EGFR expression. KRAS expression was detected in 585 lung adenocarcinoma patients and 550 lung squamous cell carcinoma patients. With BRAF expression, there were 527 lung adenocarcinoma patients and 502 lung squamous cell carcinoma patients. In lung squamous cell carcinoma and lung adenocarcinoma, EZH2 expression is positively correlated with BRAF (*r* = 0.2397, *p* < 0.0001) and KRAS (*r* = 0.3167, *p* < 0.001) gene expression (Fig. 4A, G). The EZH2 gene expression of lung adenocarcinoma was positively correlated with the gene expression of BRAF (*r* = 0.2633, *p* < 0.0001) and KRAS (r = 0.31229, p < 0.0011) (Fig. 4B, H). The EZH2 gene expression of lung squamous cell carcinoma was positively correlated with the gene expression of BRAF (*r* = 0.3662, *p* < 0.0001) and KRAS (*r* = 0.3567, *p* < 0.0001) (Fig. 4C, I). A positive correlation exists between EZH2 expression and EGFR expression in lung squamous cell carcinoma and lung adenocarcinoma, but it is weak (*r* = 0.1122, *p* = 0.0002) (Fig. 4D). However, there was no statistical significance in the statistics of lung adenocarcinoma(*r* = 0.008, *p* = 0.8594) (Fig. 4E) and lung squamous cell carcinoma(*r* = 0.0660, *p* = 0.1221) (Fig. 4F). TCGA data demonstrate a correlation between high EZH2 expression and smoking (*p* < 0.0001) (Fig. 4J), particularly in lung adenocarcinoma (*p* = 0.0011) (Fig. 4K). The result in lung squamous cell carcinoma was not statistically significant (*p* = 0.8453) (Fig. 4L).

### Survival analysis of lung cancer through Kaplan–Meier plotter database

**Fig. 5:**
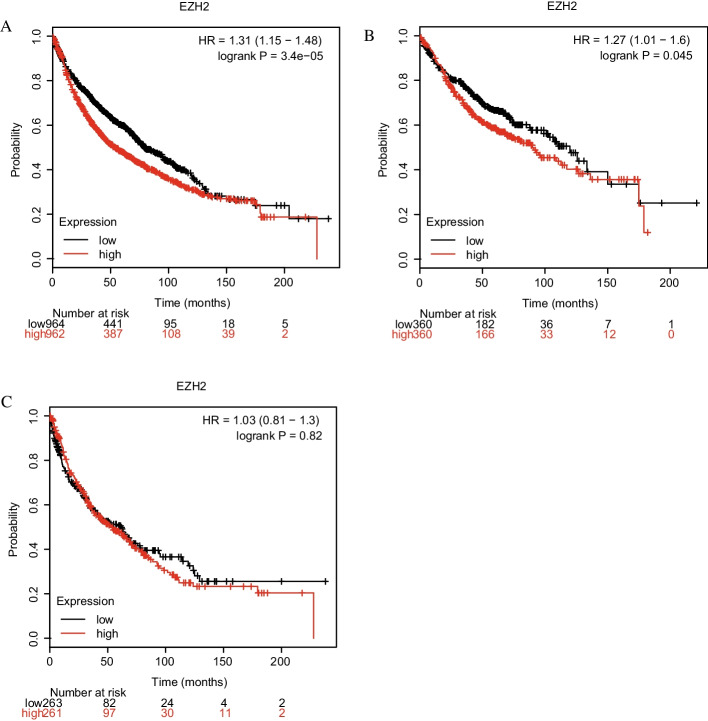
Kaplan–Meier survival curves for lung cancer patients, stratified by EZH2 expression levels. *Note*: **A** Lung cancer. **B** lung adenocarcinoma. **C** lung squamous cell carcinoma

To verify the results of this research, the Kaplan–Meier database was adopted for survival analysis. The medical records of patients diagnosed with NSCLC that were included in the study showed that high levels of EZH2 mRNA expression occurred in 962 of the cases, with a median survival time of 54.17 months; I n 964 cases with low expression, the median survival time was 79.50 months (HR = 1.31, 95% CI 1.15–1.48, *p* < 0.05). (Fig. 5A). Further analysis revealed that 360 lung adenocarcinoma cases with low expression of EZH2 had a median survival time of 119.87 months, compared to 357 cases with high expression of EZH2 (HR = 1.27, 95% CI 1.01−1.6, *p* < 0.05) (Fig. 5B). High expression of EZH2 mRNA was associated with a median survival time of 52.97 months in 261 cases of lung squamous cellcarcinoma, whereas low expression was associated with a median survival time of 62.00 months in 263 cases (HR = 1.03, 95% CI 0.81−1.3, *p* = 0:82) (Fig. 5C).

### CAMP database analysis

The CMap database was up-regulated with the top 50 EZH2-related up- and down-regulated genes from The Linked Omics database to predict potential drug targets for LUAD. These potential therapeutic targets are ranked according to a point system. CMap was applied to determine the top 15 positively correlated drugs/molecules and the top 15 negatively correlated drugs/molecules. Docetaxel, palbociclib, and angiogenesis-inhibitor were among the extensively used compounds for the treatment of tumor, as determined by their scores. Other drugs/molecules, such as MK-1775 [34, 35], MK-5108 [36], fenbendazole [37, 38], albendazole [39], BAY-K8644 [40], evodiamine [41], purvalanol-a [42], mycophenolic-acid [43], PHA-793887 [44], cyclopamine [45], is a possible treatment for lung adenocarcinoma (Tables 5, 6). To determine the therapeutic potential of these drugs/molecules in patients with lung adenocarcinoma with high EZH2 expression, additional research is necessary.

**Table 5:** The top 15 compounds with positive correlations were obtained from CMap

Rank	Score	Type	Name	Description	Target
6	99.89	cp	MLN-8054	Aurora kinase inhibitor	AURKA
10	99.68	cp	HO-013	PPAR receptor agonist	PPARG
18	99.47	cp	MK-1775	WEE1 kinase inhibitor	WEE1
20	99.4	cp	Brivanib	FGFR inhibitor	FGFR1, KDR, FLT1, CYP3A4, FGFR2, FGFR3, FLT4, KCNH2
22	99.26	cp	MK-5108	Aurora kinase inhibitor	AURKA, AURKB, AURKC
29	98.94	cp	Fenbendazole	Tubulin inhibitor	CYP2C19, CYP2D6, CYP2J2, CYP3A4, TUBB
30	98.9	cp	Cholic-acid	Bile acid	CES1, FECH, PLA2G1B, ADH1C, COX4I1, COX5A, COX5B, COX6A2, COX6B1, COX6C, COX7A1, COX7B, COX7C, COX8A, ESRRG, FABP6, GPBAR1, MT-CO1, MT-CO2, MT-CO3
33	98.8	cp	Torin-2	MTOR inhibitor	MTOR
34	98.77	cp	Docetaxel	Tubulin inhibitor	TUBB, BCL2, MAP2, MAP4, MAPT, NR1I2, TUBB1
40	98.34	cp	Albendazole	Anthelmintic	CYP1A2, CYP2J2, TUBA1A, TUBB, TUBB4B
45	97.99	cp	GANT-58	GLI antagonist	DHH, GLI1, IHH
46	97.99	cp	Buphenine	Adrenergic receptor agonist	ADRB2
52	97.85	cp	BAY-K8644	Calcium channel activator	CACNA1C
56	97.7	cp	Evodiamine	ATPase inhibitor	TRPV1
57	97.65	cp	Chaetocin	Histone lysine methyltransferase inhibitor	EHMT2, SUV39H1

*cp* compound

**Table 6:** The top 15 compounds with negative correlations were obtained from CMap

Rank	Score	Type	Name	Description	Target
8552	− 99.79	cp	Purvalanol-a	CDK inhibitor	CDK1, CDK2, CDK4, CDK5, CCND1, CCNE1, CSNK1G3, RPS6KA1, SRC
8535	− 99.5	cp	Palbociclib	CDK inhibitor	CDK4, CDK6, CCND3
8531	− 99.4	cp	JAK3-inhibitor-VI	JAK inhibitor	JAK3
8527	− 99.3	cp	BX-912	Pyruvate dehydrogenase kinase inhibitor	PDPK1, AKT2, CDK2, CHEK1, GSK3B, KDR, PDK1
8516	− 99.15	cp	Amonafide	Topoisomerase inhibitor	TOP2A, TOP2B
8513	− 99.1	cp	Mycophenolic-acid	Dehydrogenase inhibitor	IMPDH1, IMPDH2
8508	− 99.01	cp	BX-795	IKK inhibitor	PDPK1, CDK2, CHEK1, GSK3B, IKBKE, KDR, PDK1, TBK1
8503	− 98.84	cp	Aminopurvalanol-a	Tyrosine kinase inhibitor	CDK1, CDK2, CDK5, CDK6
8501	− 98.75	cp	Ellipticine	Topoisomerase inhibitor	TOP2A, TOP2B
8500	− 98.74	cp	Angiogenesis-inhibitor	Angiogenesis inhibitor	EGFR
8496	− 98.63	cp	AG-14361	PARP inhibitor	PARP1
8491	− 98.45	cp	PHA-793887	CDK inhibitor	CDK1, CDK2, CDK4, CDK5, CCND1, CCNE1, CDK7, CDK9
8488	− 98.34	cp	AS-601245	JNK inhibitor	GSK3B, MAPK10, MAPK8, MAPK9, PIM1
8489	− 98.34	cp	Cyclopamine	Smoothened receptor antagonist	SMO, DHH, IHH, PTCH1
8485	− 98.31	cp	BMS-345541	IKK inhibitor	IKBKB, CHUK

*cp* compound

## Discussion

Advanced lung adenocarcinoma is now commonly treated with immunotherapy-based combination therapy, which has demonstrated efficacy and OS advantages in the first-line metastasis. Notwithstanding the advancements in targeted therapy and immunotherapy, immune therapy patients will eventually develop drug tolerance due to immune evasion mechanisms. Patients with advanced pulmonary adenocarcinoma and metastatic lung adenocarcinoma have a dismal prognosis. In addition to PD-1/PDL1, numerous molecules, such as siglec-15 and FGL1 [46, 47], are implicated in the immune microenvironment. Consequently, investigating the latent immune-related factors of cancer immunoescape can improve the prognosis for lung adenocarcinoma patients. While the role of EZH2 in LUAD is unidentified, we sought to probe its clinical significance and biological functions by utilizing open-access databases for a comprehensive analysis.

The epigenetic modification of histones is a crucial mechanism for regulating cellular processes, such as tumorigenesis and immunity. Typically, epigenetic abnormalities are associated with tumor progression and cancer development [48]. EZH2 is the catalytic component of multicomb inhibition complex 2, which trimethylates lysine 27 of histone H3 to promote transcriptional inhibition [11]. It is frequently overexpressed in a variety of tumors, including pulmonary carcinoma [12], colonic and rectal carcinoma [49], mammary carcinoma [50], pancreatic carcinoma [47], and prostate carcinoma [47]. Multiple cancer types, including BLCA, BRCA, CESC,CHOL, COAD, ESCA, GBM, HNSC, HNSC-HPV+. KIRC,KIRP,LIHC, LUAD, LUSC, PRAD, READ, STAD, THCA, and UCEC, had elevated levels of EZH2 relative to normal tissue, according to the findings of this study (Fig. 1A, B). Our study found higher levels of EZH2 mRNA and protein in lung adenocarcinoma than in adjacent tissues. Moreover, a higher level of EZH2 expression is associated with an inferior prognosis for lung adenocarcinoma patients.

To delve deeper into the biological information of EZH2, GO and KEGG analysis is performed.These results demonstrate that the net of EZH2 expressing significantly affects the immune microenvironment in LUAD, and that the net of EZH2 expressing is essential for the onset and progression of cancers. Hoxha et al. believed that the Hippo–YAP pathway was closely related to EZH2. It may participate in this signaling pathway to inhibit the transcription of a large gene network and mediate a variety of cellular functions. This includes the inhibition of the cell cycle kinase inhibitor p27, which promotes contact inhibition and regulates the occurrence and progression of tumor cells. Moreover, EZH2 is involved in cell proliferation and organ size regulation [51]. EZH2 and JMJD6 gene profiles overlap in breast cancers, with EZH2 co-regulating a unique gene box in both ER+ and ER− cells. In MDA MB 231 cells, 496 genes, including aurora kinase, are co-regulated, and aurora kinase is currently being evaluated as a potential new treatment target for mammary carcinoma.

The CMap database was to predict potential drug targets for LUAD. And these potential therapeutic targets are ranked according to a point system. Docetaxel, palbociclib, and angiogenesis-inhibitor were among the extensively used compounds for the treatment of tumor, as determined by their scores. We have identified some potential drugs or molecules for the treatment of lung adenocarcinoma by reading the literature, such as MK-1775 [34, 35], MK-5108 [36], fenbendazole [37, 38], albendazole [39], BAY-K8644 [40], evodiamine [41], purvalanol-a [42], mycophenolic-acid [43], PHA-793887 [44], and cyclopamine [45] (Tables 5, 6). Of interest is the WEE1 inhibitor MK-1775, which has shown potential chemotherapy or radiotherapy sensitivity in preclinical models, particularly, although not exclusively, in p53 mutated or deficient cancer cells [52]. Several clinical trials have shown that WEE1 inhibitors can be safely used in combination with different chemotherapeutic agents as well as concurrent chemotherapy with radiation therapy. Ongoing clinical trials testing novel agents of WEE1 inhibitors, such as ATR and PAPR inhibitors and anti-PDL1 immunotherapies, are underway and could better define the role of WEE1 inhibitors in the future, in terms of efficacy in terms of good safety profile compared to monotherapy and/or standard of care, if any of the novel therapeutic combinations would show superior antitumor efficacy [53, 54]. In our study, EZH2 was highly expressed in lung cancers with positive KRAS expression, and the correlation was significant in lung adenocarcinoma (*r* = 0.3129 and *p* < 0.001) (Fig. 4H). The direct dependence of Methylenetetrahydrofolate dehydrogenase 2 (MTHFD2) and EZH2 expression on mutation-activated KRAS and their prognostic relevance in KRAS-mutated LUAD were identified in a study by Li et al. [55]. Aberrant KRAS activity renders LUAD cancer cell lines vulnerable to MTHFD2 and EZH2 inhibitors. Importantly, co-inhibition of these two factors has a synergistic effect [55]. Therefore the relationship between EZH2 and KRAS may be one of our breakthroughs for LUAD-targeted therapy, but the related research literature is limited and deserves further exploration.

EZH2 is associated with the modulation of pivotal regulatory targets and therapeutic targets in the cellular cycle [56]. These findings are in line with this study. Moreover, this study revealed that EZH2 is closely associated with drug metabolism, MHC protein complex and helicase activities, TF activities, transmembrane acceptor protein kinase activities, and immunoglobulin binding.

Selective deletion of EZH2 or inhibition of its hematopoietic activity with small molecules increases the production of the il-15 acceptor, CD122 + NK progenitors, and mature NK precursors in murine and human stem and precursor cells. These findings suggest that EZH2 modulates the development of NK cells and autoimmunity [57]. Phosphorylation of EZH2 interferes with the function of PRC2 and increases the expression of type I interferon and antigen presentation genes. The increased efficacy of anti-CTLA-4 immunotherapy and enhanced overall survival in tumor models of syngeneic mice [58, 59]. Inhibition of EZH2 can direct myeloid differentiation of primitive hematopoietic progenitors. Consequently, EZH2 plays a crucial yet diverse role in the modulation of TME, which is required for determination at specific phases. Our extracted data revealed that EZH2 is closely associated with immunity-related factors, such as immune stimulators, immune inhibitors, immune chemokines, and chemokine receptors. It is noteworthy that Macrophage and Neutrophil are closely associated with the expression level of EZH2. Higher inflammatory markers are associated with unsatisfactory prognoses in NSCLC patients, according to a paper we just published [60]; these results imply that the effects of EZH2 on lung adenocarcinoma may be tightly linked to inflammatory factors. Hence, the prognosis of NSCLC could be enhanced by regulating inflammatory markers.

This study based on various database mining techniques revealed that EZH2 is a biomarker associated with LUAD prognosis. The level of EZH2 expression is connected to the infiltration of immunocytes, immunomodulators, and chemokines and is involved in the cell cycle process. Our research is limited by the following factors: first, it is possible that the gene expression analysis based on the open-source database we examined is flawed. Accordingly, to explore the latent biological causal link between EZH2 and the interplay between tumor and immunity in LUAD, it is necessary to carry out additional in vivo or in vitro experiments. Second, the effects of EZH2 on the clinical outcomes of immune therapy remain undetermined and need to be clarified by additional clinical studies. Nonetheless, this study focuses on the immunofunction of EZH2 in the tumor microenvironment and its effect on the cell cycle.

In conclusion, these findings support the probability that EZH2 appears to assume an immunomodulatory function in lung adenocarcinoma. TME is crucial in determining the outcome and progression of tumor rejection. Increasing evidence suggests that it is essential to understand the effect of TIME on tumor genesis and development to accurately evaluate the efficacy of anticancer therapies and develop more effective treatments. Since EZH2 plays a role in numerous immune cells that may contribute to tumor immunity, it is essential to investigate how inhibition of EZH2 may affect immune cell function during tumor development, a question that remains unanswered at present.

## Conclusion

As a prognosis index and target gene in LUAD, highly expressed EZH2 can serve as a predictor of unsatisfactory prognoses. It is possible that the underlying cause is associated with the synergistic effect that KRAS, immune cell infiltration, and metabolic processes; correspondingly, these aspects necessitate further exploration.

## Data Availability

The datasets presented in this study can be found in online repositories. The names of the repository/repositories and accession number(s) can be found in the article. The TIMER (https://cistrome.shinyapps.io/timer/). UALCAN (http://ualcan.path.uab.edu/). The HPA (https://www.proteinatlas.org/). The Linked Omics data base (http://www.linkedomics.org/login.php). The TISIDB data base (http://cis.hku.hk/TISIDB). The CMap (https://clue.io/). The Kaplan–Meier plotter database (https://kmplot.com). TCGA data base (https://portal.gdc.cancer.gov/).

## Abbreviations

EZH2: The prognostic value of enhancer homolog 2
TCGA: The Cancer Genome Atlas
OS: Overall Survival
DSS: Disease Free Survival
PFI: Progression-Free Interval
NES: Normalized Enrichment Score
PRC2: Polycomb repressive complex 2
